# Preliminary clinical evaluation of cefmetazole dosing regimens in Japanese patients with urinary tract infections

**DOI:** 10.1186/s40780-025-00535-1

**Published:** 2025-12-27

**Authors:** Yuna Sadaka, Kokoro Nakajima, Yuki Sasaki, Mao Tsurugaya, Yasuhisa Oida, Midori Soda, Tomoyuki Hirashita, Kotoe Inoue, Yoshito Takahashi, Tsuyoshi Yokoi, Kiyoyuki Kitaichi

**Affiliations:** 1 Laboratory of Pharmaceutics, Department of Biomedical Pharmaceutics, Gifu Pharmaceutical University, 1-25-4 Daigaku-nishi, Gifu, 501-1196 Japan; 2 Department of Pharmacy, Gifu Prefectural General Medical Center, 4-6-1, Noisshiki, Gifu, 500-8717 Japan; 3 Functional Food Development for Health Promotion, Gifu Pharmaceutical University, 1-25-4 Daigaku-nishi, Gifu, 501-1196 Japan; 4 Department of Urology, Gifu Prefectural General Medical Center, 4-6-1, Noisshiki, Gifu, 500-8717 Japan

**Keywords:** Cefmetazole, HPLC, Urinary tract infection, Renal function

## Abstract

**Background:**

Cefmetazole (CMZ) is widely used in Japan to treat infections such as urinary tract infections (UTIs), intra-abdominal infections, and bloodstream infections. However, pharmacokinetic and pharmacodynamic (PK/PD) data supporting the appropriate use of CMZ in Japanese patients with UTIs remain limited. Thus, as a preliminary study, we evaluated steady-state plasma concentrations of CMZ in Japanese patients with mild to moderate UTIs.

**Methods:**

Twenty-three patients with mild to moderate UTIs admitted to the Department of Urology at Gifu Prefectural General Medical Center from October 2022 to January 2023 were enrolled. Two patients were excluded due to inappropriate sampling timing and lack of steady-state samples, resulting in 21 patients included in the final analysis. Repeated doses of cefmetazole (CMZ, 1 g-per dose) were administered at various intervals (6, 8, 12, or 24 h) based on renal function. Steady-state plasma samples 2–3.25 h after infusion (near peak) as well as just before the next dose (near trough) were collected. Plasma concentrations of CMZ were measured using a validated high-performance liquid chromatography (HPLC) method. The relationship between plasma concentrations of CMZ and creatinine clearance (Ccr) was analyzed.

**Results:**

CMZ effectively treated ESBL-E-induced UTIs in all patients (*n* = 5). With CMZ dosing regimens based on renal function, the mean (± SD) steady-state plasma concentrations at near-peak and near-trough were 73.8 ± 27.1 µg/mL and 14.0 ± 11.0 µg/mL, respectively. Steady-state near-peak plasma concentrations of CMZ (*n* = 19) were found to be negatively correlated with Ccr (*R* = − 0.70, *p* < 0.001). However, there is no correlation between Ccr and near-trough plasma concentrations of CMZ (*n* = 11, *R* = − 0.53).

**Conclusions:**

Preliminary clinical data suggest the potential appropriateness of the CMZ dosing regimen in patients with mild to moderate UTIs based on renal function, although further detailed population PK/PD analysis would be necessary.

**Supplementary Information:**

The online version contains supplementary material available at 10.1186/s40780-025-00535-1.

## Background

Recently, infections caused by extended-spectrum β-lactamase-producing Enterobacterales (ESBL-E), which are resistant to third-generation cephalosporins, have become a global health concern due to their increasing prevalence [1, 2]. Cefmetazole (CMZ), a cephamycin antibiotic, is widely used in Japan to treat infections such as urinary tract infections (UTIs), intra-abdominal infections, and bloodstream infections [3, 4]. CMZ is considered a potential treatment option for ESBL-E-induced infection because of its antibacterial activity against ESBL-producing bacteria [5]. Considering the high urinary excretion of CMZ as the unchanged form [6], CMZ would be suitable for treating UTIs, including ESBL-E-induced UTIs. Notably, several studies have demonstrated the clinical efficacy of CMZ against ESBL-E-induced UTIs in Japanese patients [7–9].

However, the international guidelines [10, 11] have not recommended the use of CMZ for ESBL-E-induced UTIs, probably due to a lack of evidence. Although a few studies have addressed the pharmacodynamic (PD) properties of CMZ [12, 13], there is still no pharmacokinetic/pharmacodynamic (PK/PD)-based evidence demonstrating its clinical utility in patients with ESBL-E-induced UTIs. Moreover, although previous PK studies examined CMZ concentrations after a single infusion [14–16], steady-state concentrations after multiple infusions would be suitable to conduct PK/PD studies in patients with impaired renal function [14].

Population pharmacokinetic (PPK) models of CMZ have varied between studies [17–19], suggesting the importance of PPK/PD analysis for CMZ in different populations. For example, plasma concentrations of CMZ in patients with critical ill or severe infection were affected by changing the volume of extracellular fluid [17] since CMZ has a relatively smaller volume of distribution [14]. Taken together, therapeutic drug monitoring for CMZ would be necessary to perform PPK/PD analyses using time above minimum inhibitory concentration (TAM), a PK/PD target for CMZ [20], to optimize dosing strategies of CMZ in patients with UTIs.

In this preliminary study, we tried to evaluate the relationship between steady-state plasma concentrations of CMZ and renal function in patients with mild to moderate UTIs treated with different dosing regimens of CMZ based on renal function.

## Methods

### Patients enrolled

This study included patients with mild to moderate UTIs treated with CMZ and admitted to the Department of Urology at Gifu Prefectural General Medical Center (GGMC) from October 2022 to January 2023. “Patients with mild to moderate UTI” were defined as patients who, assessed by physician, were diagnosed with UTI, but not critically ill, and were hemodynamically stable without systemic involvement other than fever. The exclusion criteria were as follows: (i) patients who did not provide informed consent, (ii) patients younger than 15 years, (iii) those with a urinary bacterial count < 10^5^ CFU/mL, (iv) patients undergoing renal replacement therapy (hemodialysis or peritoneal dialysis) or with creatinine clearance (Ccr) < 10 mL/min, (v) patients receiving CMZ for perioperative antimicrobial prophylaxis, and (vi) those deemed unfit for inclusion by physicians.

All patients received 1 g of CMZ via 1-h infusion, with dosing intervals adjusted according to their renal function based on our institutional regimen, which was established based on previous studies [14, 15, 21, 22], as shown in Table 1. This study was approved by the Ethics Committee of Gifu Pharmaceutical University (Approval No. 5–13) and the Ethics Review Committee of GGMC (Approval No. 758-4).

**Table 1:** Cefmetazole (CMZ) dosage regimen based on renal function

Ccr (mL/min)	> 50	30–50	10–30
Dose (g)	1	1	1
Dosing interval (h)	6–8	8–12	12–24

### Sample collections

Time points to collect steady-state blood samples were selected based on renal function since the elimination half-life (T_1/2_) was varied depending on renal function from 1 to 5.5 h [21]. Steady-state blood samples were collected 2–3.25 h after the start of infusion (near peak) and 6–24 h after the start of the final CMZ infusion (near trough). Blood samples for near-peak following CMZ administration were collected once a day, with routine collection for obtaining laboratory data. When multiple steady-state near-peak samples were available from the same patient, a later post-dose or a later-day sample was selected for analysis to reflect the elimination phase at steady-state.

Near-trough samples were collected after the final dose of CMZ, at a time corresponding to the trough based on their dosing schedule. Physicians decided the specific timing of sample collections for all samples. Blood samples were centrifuged to separate plasma and stored at − 60 °C until analysis (see details in the Supplemental Data).

### Measurement of plasma concentrations of CMZ in patients with UTIs

Plasma concentrations of CMZ were measured using high-performance liquid chromatography (see details in the Supplemental Data).

### Data analysis

The relationship between plasma concentration of CMZ and Cockcroft–Gault equation-estimated Ccr, in patients with mild to moderate UTIs was evaluated using Pearson’s product-moment correlation coefficient. A *p*-value of less than 0.05 was considered statistically significant. Statistical analyses were performed using JMP^®^ Pro (version 17.2.0; SAS Institute Inc., Cary, North Carolina, USA).

## Results and discussion

### Patient characteristics

**Fig. 1:**
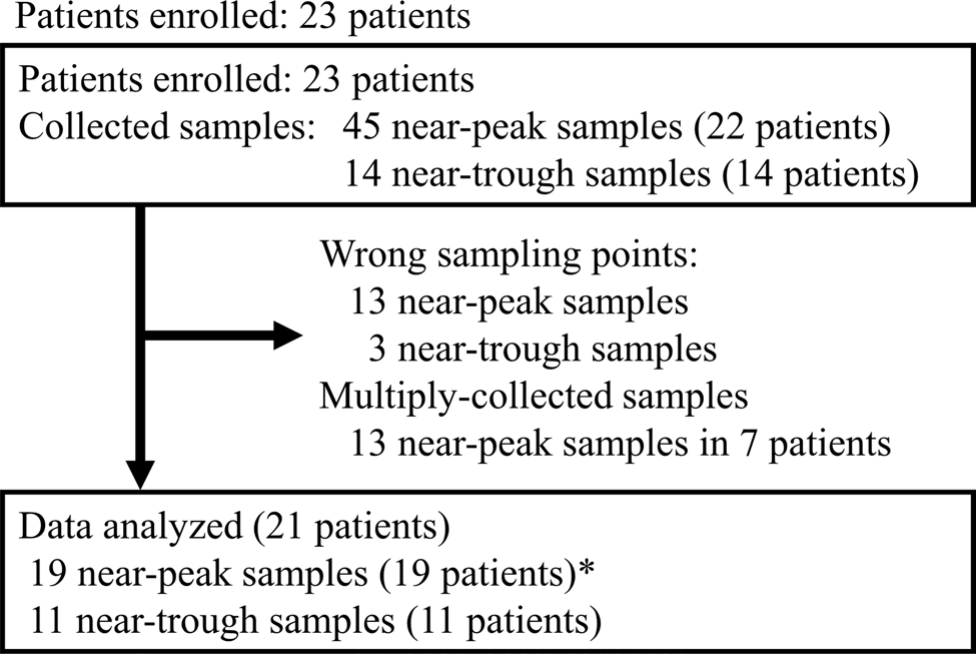
Patient enrollment and sampling summary *In patients who collected near-peak multiple samples, we selected the sample collected at the later post-dose time or on a later day

Figure 1 shows the patient enrollment and sampling summary. Twenty-three patients met the inclusion criteria. Two patients were excluded due to the sample collections before reaching the steady-state and at time points that were neither near-peak nor near-trough, respectively. Table 2 presents the characteristics of 21 patients analyzed. The average age of patients with UTIs was 77.9 ± 7.4 years. Among these patients, 66.7% were diagnosed with pyelonephritis.

**Table 2:** The characteristics of patients with mild to moderate urinary tract infections (UTIs), the selected dosing intervals of cefmetazole (CMZ), and the effect of CMZ in patients infected with Extended-spectrum β-lactamase-producing Enterobacterales (ESBL-E)

Sex (male/female, *n*)					10/11			
Age, years (mean ± SD)					77.9 ± 7.4			
Weight, kg (mean ± SD)					57 ± 12			
Laboratory values at admission					Mean ± SD			
		Scr, mg/dL			1.4 ± 0.7			
		AST, IU/L			28.9 ± 17.4			
		ALT, IU/L			21.2 ± 13.3			
		Alb, g/dL			3.3 ± 0.6			
		BUN, mg/dL			23.9 ± 10.5			
		CRP, mg/dL			13.9 ± 7.1			
		T-bil, mg/dL			1.0 ± 0.5			
		WBC, ×10³/µL			15.8 ± 9.2			
		Ccr, mL/min			41.1 ± 20.7			
					n (%)			
		> 50			5 (23.8)			
		30–50			9 (42.9)			
		10–30			7 (33.3)			
Complicating diseases					n (%)			
		Hyperlipidemia			13 (61.9)			
		Hypertension			12 (57.1)			
		Diabetes			6 (28.6)			
Concomitant medications					n (%)			
		Lipid-lowering agents			13 (61.9)			
		Antihypertensive agents			12 (57.1)			
		Gastric secretion inhibitors			11 (52.4)			
		Hypoglycemic agents			5 (23.8)			
Types of infection					n (%)			
		Pyelonephritis			14 (66.7)			
		Prostatitis			4 (19.0)			
		Postoperative UTI			2 (9.5)			
		Renal cyst infection			1 (4.8)			
Bloodstream infection					n (%)			
					12 (57.1)			
Bacterial species					No. of isolates, n *^1^			
					Urine		Blood	
		**ESBL producing** —						
		Enterobacterales *–Escherichia coli* *–Klebsiella pneumoniae* Non-Enterobacterales			5 4 1 0		3 3 0 0	
		**Non-ESBL producing** —						
		Enterobacterales *–Escherichia coli* *–Klebsiella pneumoniae* *–*others *^2^ Non-Enterobacterales			17 9 3 5 8		9 4 2 3 2	
The selected dosing intervals of CMZ								
		q6 h (n)	q8 h (n)	q12 h (n)		q24 h (n)		
Ccr, mL/min	> 50	2	3	0		0		
	30–50	1*^3^	4	4		0		
	10–30	0	1*^3^	6		0		
The efficacy of CMZ in patients infected with ESBL-E*^4^								
		Cured		Not cured				
		5		0				

AST; Aspartate transaminase, ALT; Alanine transaminase, Alb; Albumin, BUN; Blood urea nitrogen, Ccr; Creatinine clearance, CRP; C-reactive protein, ESBL; Extended-spectrum β-lactamase, Scr; Serum creatinine, T-bil; Total bilirubin, WBC; White blood cells

*^1^ Several patients had experienced polymicrobial infections

*^2^ “Others” includes *Klebsiella variicola*, *Klebsiella oxytoca*, *Proteus mirabilis*, and *Citrobacter farmeri*

*^3^ Physicians decided to administer CMZ with these intervals, expecting the quick improvement of renal function

*^4^ The efficacy of CMZ was evaluated only in patients infected with ESBL-E, since the de-escalation and other interventions were performed in patients with non-ESBL-E. “Cure” was defined as physician-judged successful treatment of UTIs with CMZ

### Standard curve and typical chromatograms of CMZ in patient samples

**Fig. 2:**
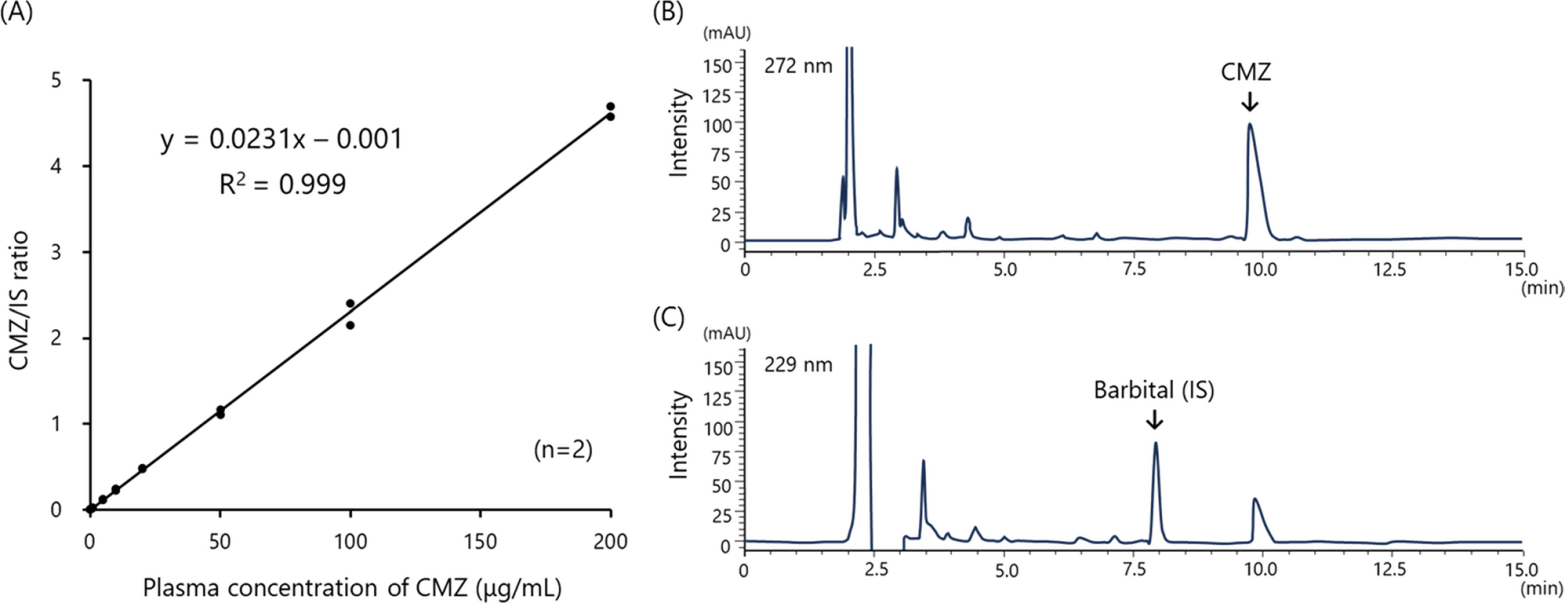
(**A**) Standard calibration curve of cefmetazole (CMZ) and representative chromatogram of CMZ (**B**) and internal standard (IS) (**C**) in patients with mild to moderate urinary tract infections (UTIs)

Figure 2A shows the standard curve of CMZ in pooled human plasma spiked with CMZ. The curve demonstrated good linearity (R^2^ = 0.999) over the concentration range of 0–200 µg/mL. Figure 2B and C show typical chromatograms from a patient sample. No significant interfering peaks were observed in the retention time regions of both CMZ and internal standard (IS), indicating the method’s specificity (Fig. 2B and C). There were no interfered peaks in the chromatograms of all patients (data not shown) despite the use of multiple concomitant medications (Table 2).

### The relationship between renal function, steady-state plasma concentrations of CMZ, and clinical efficacy of CMZ

We used 19 steady-state near-peak samples from 19 patients and 11 steady-state near-trough samples from 11 patients for the analysis (Fig. 1). Notably, the dosing regimens of CMZ based on renal function effectively treated ESBL-E-induced UTIs in all patients (*n* = 5; Table 2).

**Fig. 3:**
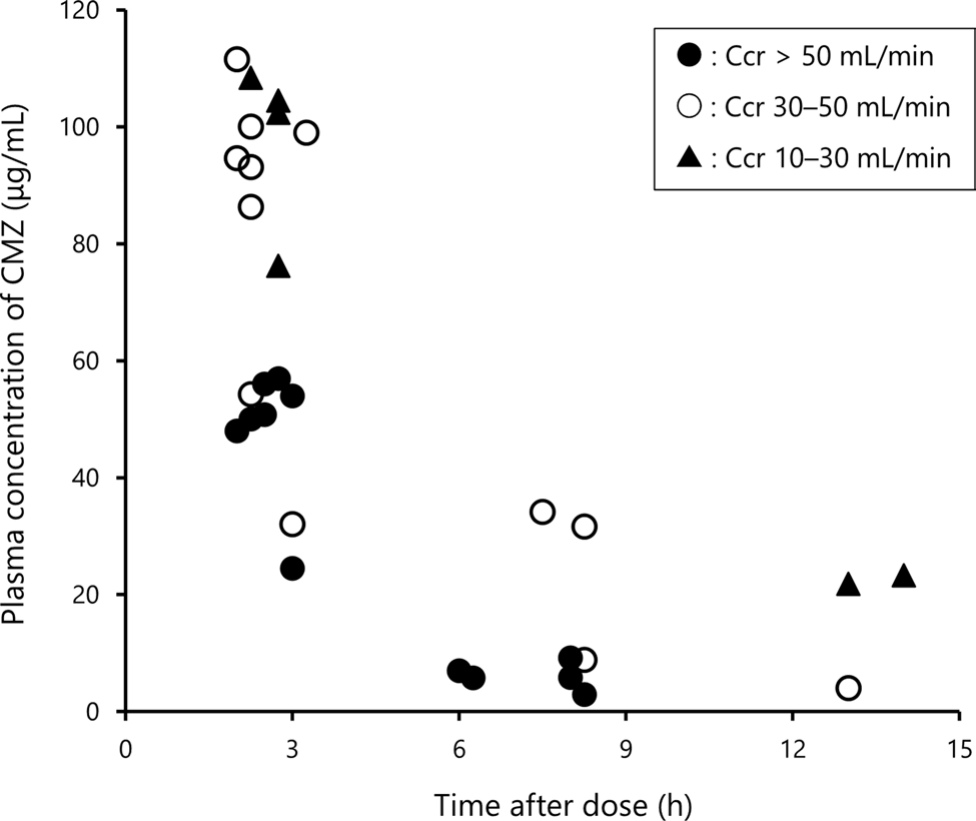
Relationship between time after the start of infusion and plasma concentrations of CMZ in patients with mild to moderate UTIs. Closed circles represent patients with creatinine clearance (Ccr) > 50 mL/min; open circles indicate those with Ccr 30–50 mL/min; and closed triangles represent those with Ccr 10–30 mL/min. The values of Ccr used were obtained at the time of blood sampling

Figure 3 shows the relationship between time after dose of CMZ and steady-state plasma concentrations of CMZ. Plasma concentrations of CMZ were time-dependently decreased (Fig. 3). Steady-state plasma concentrations of CMZ at near-peak and near-trough (mean ± SD) were 73.8 ± 27.1 µg/mL and 14.0 ± 11.0 µg/mL, respectively.

**Fig. 4:**
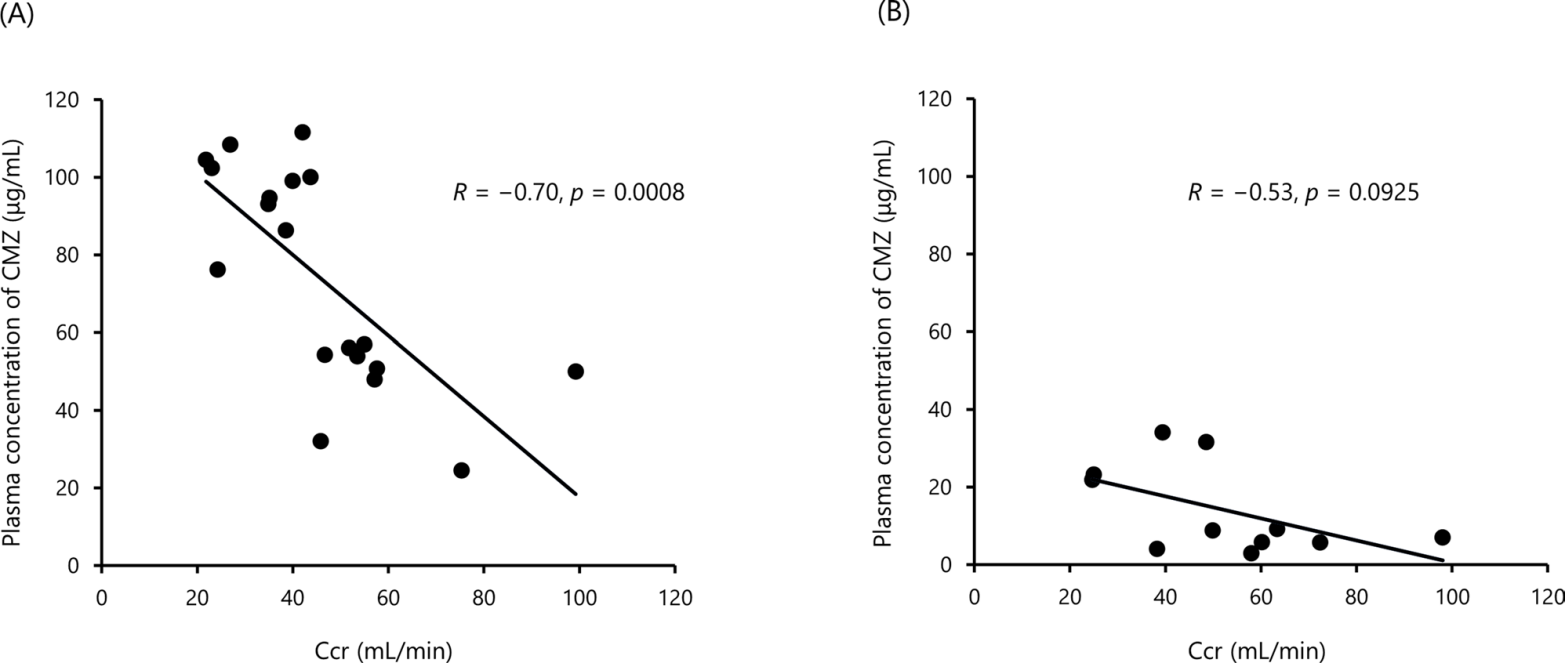
Relationship between Ccr and plasma concentrations of CMZ at (**A**) near-peak (*n* = 19) and (**B**) near-trough (*n* = 11) in patients with mild to moderate UTIs. The values of Ccr used were obtained at the time of blood sampling

Steady-state near-peak plasma concentrations of CMZ (*n* = 19) were negatively correlated with Ccr (*R* = − 0.70, *p* = 0.0008) (Fig. 4A). Our findings are partially consistent with a previous report demonstrating that peak plasma concentrations of CMZ were higher in patients with impaired renal function compared to healthy subjects following a single infusion [15]. Considering the fact that the impaired renal function did not alter the volume of distribution of CMZ at steady-state [14], the reduced renal clearance of CMZ prolongs the T_1/2_ of CMZ, subsequently elevating steady-state near-peak concentrations of CMZ.

Instead, steady-state near-trough plasma concentrations of CMZ (*n* = 11) did not show a significant correlation with renal function (*R* = − 0.53, *p* = 0.0925) (Fig. 4B). The lack of a significant correlation between renal function and near-trough concentrations of CMZ may reflect the appropriateness of CMZ dosing regimen.

In seven patients, the steady-state near-trough concentrations of CMZ were below 10 µg/mL (Fig. 4B). Among them, five patients had high Ccr (> 50 mL/min) together with relatively lower steady-state near-peak concentrations (< 60 µg/mL, Fig. 3). Although higher or more frequent dosing may be required in patients with high Ccr, CMZ successfully treated UTIs in all such patients with high Ccr (two ESBL-E and three non-ESBL-E). The remaining two patients were also cured with CMZ. The minimum inhibitory concentrations (MIC)₉₀ of CMZ (4 µg/mL) and MICs in most isolates (≤ 8 µg/mL) against ESBL-E in Japan were all below 10 µg/mL [17]. Taken together, these results again suggest the appropriateness of the CMZ dosing regimen, although we did not measure MICs.

No adverse events leading to discontinuation of CMZ were observed (data not shown). Moreover, no patients in this study received drugs inhibiting renal tubular secretion of CMZ such as probenecid [23]. Furthermore, the type of infection (with or without bloodstream infection) and bacterial species such as ESBL-E did not affect the plasma concentrations of CMZ (data not shown).

### Limitations of the study

The number of samples was limited, which may affect the robustness of the observed correlation between Ccr and plasma concentrations of CMZ. Therefore, further studies with larger sample sizes are needed to validate these findings, together with PPK/PD analysis.

## Conclusion

CMZ dosing regimens based on renal function maintain steady-state near-trough concentrations of CMZ within a similar range, and CMZ was clinically effective against ESBL-E-induced UTIs. Thus, our preliminary study suggested the appropriateness of CMZ dosing regimens in patients with mild to moderate UTIs, including ESBL-E-induced UTIs. Further PPK/PD studies are warranted for individually optimizing CMZ dosing regimens.

## Supplementary Information

Below is the link to the electronic supplementary material.

Supplementary Material 1

## Data Availability

The data supporting the findings of this study are available upon reasonable request.

## Abbreviations

Ccr: Creatinine clearance
CMZ: Cefmetazole
ESBL-E: Extended-spectrum β-lactamase-producing Enterobacterales
GGMC: Gifu Prefectural General Medical Center
HPLC: High-performance liquid chromatography
IS: Internal standard
MIC: Minimum inhibitory concentration
PK: Pharmacokinetic
PD: Pharmacodynamic
PPK: Population pharmacokinetics
T_1/2_: Elimination half-life
TAM: Time above minimum inhibitory concentration
UTI: Urinary tract infection
