# Supplementary material for: Preliminary clinical evaluation of cefmetazole dosing regimens in Japanese patients with urinary tract infections
Source: J Pharm Health Care Sci. 2025 Dec 27;12:12. doi: 10.1186/s40780-025-00535-1 (PMC12853748; doi:10.1186/s40780-025-00535-1)
Supplement: Supplementary file 1 — Supplementary Material 1 [file 40780_2025_535_MOESM1_ESM.docx]

**Preliminary Clinical Evaluation of Cefmetazole Dosing Regimens in Japanese Patients with Urinary Tract Infections**

**Supplemental Data**

**Materials and Methods**

***Chemicals and reagents***

Cefmetazole sodium (purity 98%) was purchased from SIGMA-Aldrich Co., LLC (St. Louis, USA). Barbital sodium (purity > 98%) was purchased from Tokyo Kasei Co., Ltd. (Tokyo, Japan). Distilled water and acetonitrile (MeCN) for HPLC grade were purchased from Hikari-Pharm Co., Ltd. (Osaka, Japan) and FUJIFILM Wako Co., Ltd. (Tokyo, Japan), respectively. Human plasma used for calibration curves and validation was purchased from Cosmo Bio Co., Ltd. (Tokyo, Japan). Citric acid and trisodium citrate dihydrate used for mobile phase preparation were purchased from FUJIFILM Wako Co., Ltd. Millex^®^-LG filters (0.20 μm; Merck, Darmstadt, Germany) were used for sample filtration.

***Liquid chromatography***

HPLC analysis was performed using a SHIMADZU LC-20AD system (Shimadzu Co., Ltd., Kyoto, Japan). Samples were separated using a COSMOSIL^®^ 5C_18_-MS-II column (4.6 ID × 250 mm; Nacalai Tesque Co., Ltd., Kyoto, Japan). The column length was selected based on the previous reports (1,2). The mobile phase consisted of 5 mM sodium citrate buffer (pH 3.2) and MeCN (85:15, v/v%). The flow rate was maintained at 1.2 mL/min, and the column temperature was set at 45°C. The total run time was 15 min. The injection volume was 50 µL, and the detection wavelengths were 272 nm (CMZ) and 229 nm (IS). Initially, based on previous reports (1,3), we used 5 mM sodium citrate buffer (pH 5.4). However, the interfering peaks derived from plasma overlapped with the CMZ peak. Considering the pKa of CMZ (approximately 2.3), we lowered the mobile phase pH to increase the proportion of the molecular (non-ionized) form. As a result, we selected 5 mM sodium citrate buffer (pH 3.2) based on the separation and peak shape.

***Preparation of stock solutions***

CMZ and Barbital sodium as the internal standard (IS) were dissolved in 15% MeCN in water to final concentrations of 20 mg/mL and 1 mg/mL, respectively, and stored at −30°C until analysis. Barbital sodium was selected as IS based on the previous reports (1–3)

***Preparation of standard and*** ***quality control (QC) samples***

The CMZ stock solution was further diluted with 15% MeCN in water to prepare working solutions at various concentrations. The IS stock solution was diluted with MeCN to 40 μg/mL. Calibration standards and QC samples were prepared by diluting the corresponding working solutions with blank human plasma. Final calibration standard concentrations were 0, 0.5, 1, 5, 10, 20, 50, 100 and 200 μg/mL. Calibration curve was constructed over the concentration range of 0–200 μg/mL.

***Preparation of samples to measure plasma CMZ***

Samples were centrifuged at 12,500 rpm for 10 min at 4°C. The supernatant was dried completely under a nitrogen stream. The residue was reconstituted with 200 µL of mobile phase, filtered, and 50 µL was injected into the HPLC system.

***Determination of plasma CMZ concentration***

Calibration curves were generated by plotting the peak area ratio (CMZ/IS) against nominal concentrations. CMZ concentrations in plasma were calculated using the regression equation derived from the calibration curve.

**Method validation**

The method was validated by FDA guidance for bioanalytical method validation (4). Accuracy was acceptable when the relative error (RE) was within ±15%, and precision was acceptable when the relative standard deviation (RSD) was ≤15%, except at the lower limit of quantification (LLOQ), where RE ≤ ±20% and RSD ≤ 20%.

***Selectivity***

Selectivity was evaluated by comparing chromatograms of blank plasma and plasma containing 200 µg/mL CMZ. No interference was observed in the CMZ and IS retention time regions.

***Intra-day accuracy and precision***

QC samples for accuracy and precision assessment were defined as follows: LLOQ at 0.5 µg/mL, low QC (LQC) at 5 µg/mL, medium QC (MQC) at 10, 20, and 100 µg/mL, and high QC (HQC) at 200 µg/mL. QC samples were measured five times within a single day. Accuracy and precision were calculated to assess within-run reproducibility.

***Inter-day accuracy and precision***

QC samples were measured once daily for five days. Accuracy and precision were calculated to evaluate between-run reproducibility.

***Recovery***

Recovery was calculated by comparing the peak area ratios of (a) plasma samples spiked with CMZ and processed before analysis, and (b) blank plasma processed first and then spiked with CMZ, using the following formula:

Recovery (%)＝$\frac{a}{b}$ × 100

***Stability***

The stability of CMZ was evaluated in QC samples at four concentrations including LQC (5 μg/mL), MQC (10 and 100 μg/mL) and HQC (200 μg/mL). HPLC measurements were performed in triplicate under the following conditions:

***Freeze-thaw stability***

The stability of CMZ in plasma samples subjected to repeated freeze-thaw cycles—common during sample storage—was assessed. QC samples at four concentrations were frozen at −30°C for 24 h and then thawed at room temperature. This freeze-thaw cycle was repeated three times. Samples were then prepared and analyzed using the method described above.

***Bench-top stability***

To assess stability under typical sample preparation conditions, QC samples (CMZ-containing plasma) were left at room temperature for 24 h and then processed using the aforementioned method.

***Short-term stability***

Short-term stability of CMZ in plasma was assessed by analyzing CMZ-containing plasma stored at −30°C for one week. Samples were processed and analyzed according to the aforementioned method.

***Long-term stability***

Long-term stability was assessed by analyzing CMZ-containing plasma stored at −30ºC for two months. Additionally, CMZ stock solutions were evaluated for stability under conditions relevant to long-term storage.

***Autosampler stability***

QC plasma samples containing CMZ were prepared using the aforementioned method and left in an autosampler at room temperature for 24 h. The stability of the samples was assessed by measuring CMZ concentrations from the time of preparation until analysis.

**Results and Discussion**

***Method validation***

*Selectivity and linearity*

Fig. S1 illustrates typical chromatograms of plasma samples containing CMZ and IS, as well as blank plasma samples without CMZ and IS. CMZ and IS were detected at retention times of 9.9 and 7.9 min, respectively. No significant interfering peaks were observed in the CMZ and IS peak regions. The proposed method requires approximately 15 min per sample, which is consistent with previous reports (3).

The calibration curve demonstrated good linearity in the plasma concentration range of 0–200 μg /mL (R^2^ = 0.999) (Fig. 2A). The average maximum plasma concentration (C_max_) after a 1 g intravenous infusion of CMZ over 1 h in healthy adults is approximately 76.2 μg/mL, and the half-life (T_1/2_) is approximately 1.1 h (5). In patients with renal dysfunction (Ccr ≤ 70 mL/min), plasma CMZ concentrations are elevated, and T_1/2_ is prolonged (5). Another report noted that T_1/2_ in patients with Ccr between 10–39 mL/min was approximately 6 h (6). Based on these data, the C_max_ at steady state (after the fifth dose of 1 g administered twice daily) was estimated to reach approximately 130 μg/mL. Therefore, the upper limit of the calibration curve was set at 200 μg/mL.

The LLOQ in the proposed method is 0.5 μg/mL, consistent with previous reports (2,3,7). Theoretical plate number and symmetry factor the QC sample at a plasma concentration of 50 μg/mL were 7842 and 2.3, respectively.

Among 43 samples, five samples were >100 μg/mL and no sample were <0.5 μg/mL.

***Accuracy and precision***

The intra- and inter-day accuracy and precision of CMZ plasma measurements met the criteria outlined in the FDA guidance. The RE was within ±11%, and RSD was <11% for all QC samples (Supplemental Table 1). The RSD for intra- and inter-day reproducibility ranged from 1.2% to 10.5%, which is below the FDA acceptance threshold of 15% (4), indicating that the proposed method has high reproducibility (Supplemental Table 1). It should be noted that, according to FDA guidance (4), the LQC should ideally be set within three times the LLOQ; however, in this study, it was established at 5 µg/mL. Since no trough concentrations below 0.5 µg/mL were detected in actual clinical samples, evaluating stability under various conditions at LQC of 5 µg/mL was considered sufficient and meaningful for measurements following multiple dosing.

*Recovery*

The recovery rates of CMZ in plasma were greater than 94.3% across all six concentrations, with minimal variability between concentrations (Supplemental Table 1).

*Stability*

Supplemental Table 2 summarizes the results of stability analyses under various storage and handling conditions for CMZ in plasma. The RE and RSD values for CMZ in plasma remained within 15% for all tested conditions, including bench-top stability (24 h at room temperature) and both short-term (1 week) and long-term (2 months) stability at −30°C. After three freeze-thaw cycles, RE and RSD were within 6%, indicating that CMZ stability was not affected by repeated freezing and thawing. Moreover, the autosampler stability results also indicated reliable post-preparative stability, with RE and RSD within 7%. All stability tests met the FDA guidance acceptance criteria for accuracy (≤ ±15%) and precision (≤15%) (4). Additionally, the CMZ stock solution was stable at −30°C for up to 3 months.

***Additional information***

To accommodate facilities using single-wavelength detectors, we also evaluated the quantification accuracy using an external calibration curve (without an IS). Good linearity was achieved (R² = 0.999), and the RE values for all QC concentrations on the calibration curve were confirmed to be within ±15% (data not shown).

**References**

1. Ko H, Novak E, Peters G. R, Bothwell W. M, Hosley J. D, Closson S. K, et al. Pharmacokinetics of single-dose cefmetazole following intramuscular administration of cefmetazole sodium to healthy male volunteers. Antimicrob Agents Chemother. 1989;33:508–512.
2. Welage LS, Borin MT, Wilton JH, Hejmanowski LG, Wels PB, Schentag JJ. Comparative evaluation of the pharmacokinetics of N-methylthiotetrazole following administration of cefoperazone, cefotetan, and cefmetazole. Antimicrob Agents Chemother. 1990;34:2369-74.
3. Sekine M, Sasahara K, Kojima T, Morioka T. High-performance liquid chromatographic method for determination of cefmetazole in human serum. Antimicrob Agents Chemother. 1982;21:740-3.
4. U.S. Department of Health and Human Services, Food and Drug Administration Center for Drug Evaluation and Research, Center for Veterinary Medicine, Department of Health and Human Services. Bioanalytical method validation guidance for industry. 2018. https://www.fda.gov/regulatory-information/search-fda-guidance-documents/bioanalytical-method-validation-guidance-industry.
5. Kawada Y. Profile of cefmetazole. II. Absorption, excretion, distribution and metabolis. Kansenshogaku Zasshi. 1979;53:66-74.
6. Halstenson CE, Guay DR, Opsahl JA, Hirata CA, Olanoff LS, Novak E, et al. Disposition of cefmetazole in healthy volunteers and patients with impaired renal function. Antimicrob Agents Chemother. 1990;34:519-23.
7. Tomizawa A, Nakamura T, Komatsu T, Inano H, Kondo R, Watanabe M, et al. Optimal dosage of cefmetazole for intraoperative antimicrobial prophylaxis in patients undergoing surgery for colorectal cancer. J Pharm Health Care Sci. 2017;3:1.

**Figure legends for supplemental data**

**Fig. S1 Typical chromatograms of CMZ (200 μg/mL) and IS (40 μg/mL) in plasma and blank plasma.**

(a) CMZ-spiked plasma (detected at 272 nm); (b) IS-spiked plasma (at 229 nm); (c) blank plasma (at 272 nm), (d) blank plasma (at 229 nm).

**Fig. S1, Sadaka Y. et al.**


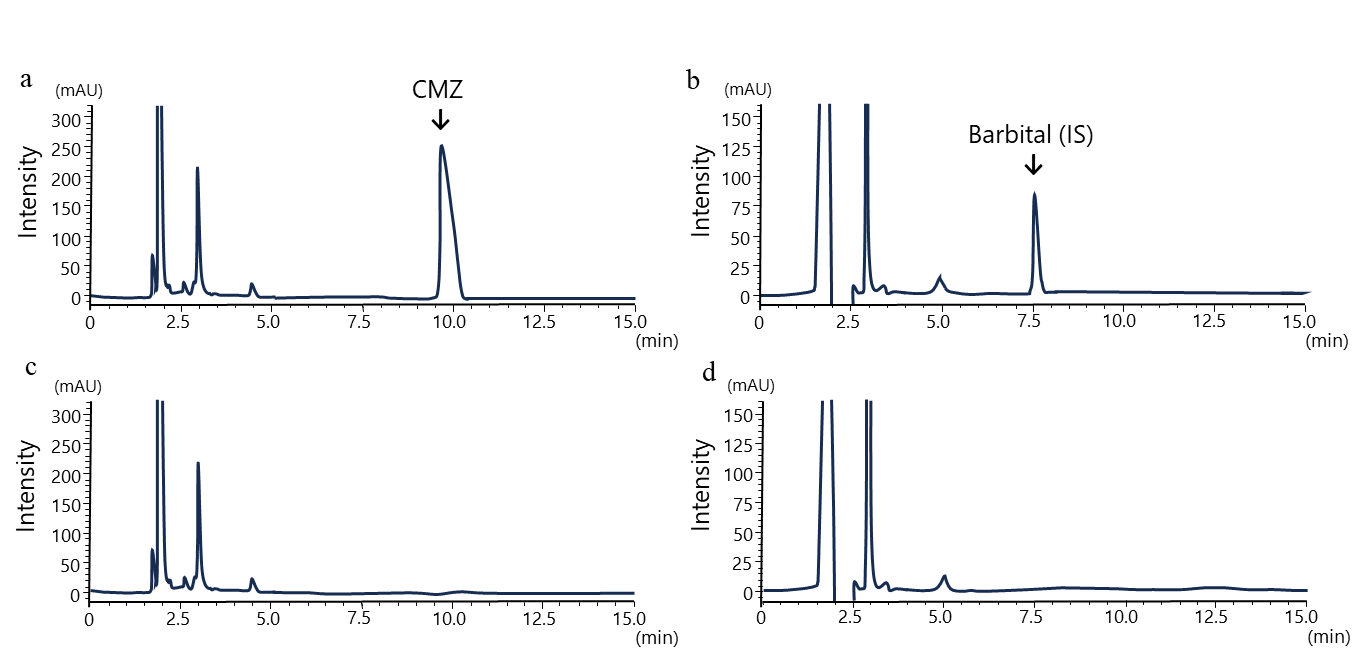


**Supplemental Table 1 Intra- and inter-day precision, accuracy, and recovery for determination of CMZ in plasma**

| Added C | Intra-day (n=5) | | |  | Inter-day (n=5) | | |  | Recovery |
| --- | --- | --- | --- | --- | --- | --- | --- | --- | --- |
|  | Found C | RSD | RE |  | Found C | RSD | RE |  |  |
| (μg/mL) | (μg/mL) | (%) | (%) |  | (μg/mL) | (%) | (%) |  | (%, n=3) |
| 0.5 | 0.49 ± 0.05 | 10.51 | −1.40 |  | 0.54 ± 0.03 | 4.88 | 8.79 |  | 112.96 ± 4.65 |
| 5 | 4.47 ± 0.19 | 4.31 | −10.50 |  | 5.06 ± 0.30 | 5.93 | 1.20 |  | 102.68 ± 7.53 |
| 10 | 8.93 ± 0.32 | 3.63 | −10.72 |  | 10.09 ± 0.56 | 5.51 | 0.86 |  | 94.25 ± 3.91 |
| 20 | 18.97 ± 0.37 | 1.95 | −5.15 |  | 19.83 ± 1.54 | 7.76 | −0.85 |  | 102.03 ± 2.65 |
| 100 | 94.31 ± 2.80 | 2.97 | −5.69 |  | 100.57 ± 5.10 | 5.08 | 0.57 |  | 99.29 ± 5.47 |
| 200 | 196.65 ± 2.37 | 1.20 | −1.67 |  | 208.01 ± 10.68 | 5.13 | 4.00 |  | 101.03 ± 6.26 |

RSD : Precision, RE : Accuracy, Found C and recovery: Mean ± SD.

**Supplemental Table 2 Effect of storage conditions on the stability of CMZ in plasma**

|  | RSD (%, n=3) | | | |  | RE (%, n=3) | | | | | | |
| --- | --- | --- | --- | --- | --- | --- | --- | --- | --- | --- | --- | --- |
|  | CMZ concentration (μg/mL) | | | |  | CMZ concentration (μg/mL) | | | | | | |
|  | 5 | 10 | 100 | 200 |  | 5 | 10 | | 100 | | | 200 |
| Bench-top stability | 4.14 | 0.92 | 2.02 | 1.57 |  | 5.68 | | 9.16 | | 10.74 | 9.86 | |
| Autosampler stability | 1.70 | 2.69 | 1.86 | 1.62 |  | 4.24 | | 6.77 | | 3.36 | 0.17 | |
| Short-term stability | 1.49 | 2.84 | 0.95 | 1.35 |  | 13.60 | | 14.62 | | 11.74 | 12.59 | |
| Long-term stability | 1.31 | 1.29 | 0.71 | 3.44 |  | −10.79 | | −8.60 | | −14.46 | −12.46 | |
| Freeze/thaw stability | 0.41 | 5.94 | 3.63 | 1.73 |  | −1.70 | | −1.28 | | −2.58 | 2.07 | |

RSD : Precision, RE : Accuracy.
